# Adiponectin receptor agonist AdipoRon blocks skin inflamm‐ageing by regulating mitochondrial dynamics

**DOI:** 10.1111/cpr.13155

**Published:** 2021-11-01

**Authors:** Jiachen Sun, Xinzhu Liu, Chuan’an Shen, Wen Zhang, Yuezeng Niu

**Affiliations:** ^1^ Department of Burns and Plastic Surgery The Fourth Medical Center of Chinese PLA General Hospital Beijing China

**Keywords:** AdipoRon, Dynamin‐related protein 1 (Drp1), inflamm‐ageing, mitochondria, SASP, skin

## Abstract

**Introduction:**

Skin is susceptible to senescence‐associated secretory phenotype (SASP) and inflamm‐ageing partly owing to the degeneration of mitochondria. AdipoRon (AR) has protective effects on mitochondria in metabolic diseases such as diabetes. We explored the role of AR on mitochondria damage induced by skin inflamm‐ageing and its underlying mechanism.

**Methods:**

Western blot, immunofluorescence and TUNEL staining were used to detect inflammatory factors and apoptosis during skin ageing. Transmission electron microscopy, ATP determination kit, CellLight Mitochondria GFP (Mito‐GFP), mitochondrial stress test, MitoSOX and JC‐1 staining were used to detect mitochondrial changes. Western blot was applied to explore the underlying mechanism. Flow cytometry, scratch test, Sulforhodamine B assay and wound healing test were used to detect the effects of AR on cell apoptosis, migration and proliferation.

**Results:**

AR attenuated inflammatory factors and apoptosis that increased in aged skin, and improved mitochondrial morphology and function. This process at least partly depended on the suppression of dynamin‐related protein 1 (Drp1)‐mediated excessive mitochondrial division. More specifically, AR up‐regulated the phosphorylation of Drp1 at Serine 637 by activating AMP‐activated protein kinase (AMPK), thereby inhibiting the mitochondrial translocation of Drp1. Moreover, AR reduced mitochondrial fragmentation and the production of superoxide, preserved the membrane potential and permeability of mitochondria and accelerated wound healing in aged skin.

**Conclusion:**

AR rescues the mitochondria in aged skin by suppressing its excessive division mediated by Drp1.

## Graphical abstract

AdipoRon can effectively activate the AMPK pathway and reverse the down‐regulation of the phosphorylation level of Drp1 at serine 637 in epidermal stem cells (ESCs) under the inflammatory environment, thus attenuate excessive mitochondrial translocation of Drp1. The repression of the mitochondrial fragments by AdipoRon protected the morphology and function of mitochondria under inflammation, thus contributed to an alleviated ROS and inflammation levels, and ultimately improved proliferation and migration of ESCs and wound healing in aged skin.

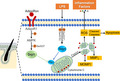

## 1 INTRODUCTION

With the development of age, the inevitable cellular senescence is driven by various factors, including mitochondrial function disorder, telomere shortening, accumulation of DNA damage and weakening of DNA repair response. Senescent cells are characterized by permanent exit from the cell cycle and degeneration of cell structure.^1^, ^2^ While temporarily existing senescent cells, which can be selectively eliminated are beneficial for obtaining a longer lifespan to a certain extent, their long‐term accumulation and persistence with age will negatively affect surrounding tissues by the secretion of pro‐inflammatory factors, collectively known as senescence‐associated secretory phenotype (SASP), including the secretion of chemokines and extracellular matrix (ECM) remodelling proteases.^3^, ^4^, ^5^ The healthier or younger cells in the aged tissue can be affected by the pro‐inflammatory factors secreted continuously by the adjacent senescent cells, causing DNA breakage and oxidative stress damage, which eventually leads to ageing‐associated inflammation and further facilitated senescence.^6^ Not like the normal or moderate inflammation, which is a defence response of the body to various stimuli, the ageing‐associated inflammation induced by SASP is chronic and aseptic, leading to a vicious circle of progressive functional and morphological abnormality in tissues and organs.^7^ This can be counteracted by: (1) ameliorating or selectively eliminating senescent cells, which is the main producer of SASP, or by (2) inhibiting the generation of reactive oxygen species (ROS) and the release of inflammatory factors.

As a multilayered organ, the skin is essential for regulating body temperature, maintaining body fluids and defending against external pathogens.^8^ Mainly located in the basal layer of the skin, epidermal stem cells (ESCs) have strong proliferation ability and can continuously produce keratinocytes through asymmetric division, thus supporting the renewal of the skin. The energy required in the constant renewal of the skin is met mainly by mitochondrial aerobic respiration.^9^ In addition to synthesizing ATP, mitochondria can also produce oxidative stress products such as ROS. Moderate ROS production has physiological effects, for example, as a molecular signal to activate the stress response that is beneficial to the organism. Yet, the overaccumulation of ROS may trigger oxidative stress and inflammation response.^10^ The oxidative damage caused by the over production of mitochondrial ROS has been identified as the molecular basis of various pathophysiological conditions, such as ageing and metabolic diseases.^11^, ^12^

Not surprisingly, influenced by both natural ageing and photoageing, ESCs are inevitably impaired by the SASP and the inflamm‐ageing in aged skin, manifested as limited proliferation and migration, eventually leading to epidermal atrophy and wound healing delay.^13^, ^14^ Tracing the underlying mechanism, mitochondria are the main organelle affected during the chronological and ultraviolet‐induced skin ageing process, accompanied by mitochondrial fragmentation and dysfunction,^15^ leading to reduced ATP production and increased ROS production. Given the above evidence, mitochondria‐targeted therapy may be the key to solving the SASP and inflamm‐ageing of the skin by enhancing the mitochondrial function of senescent cells, inhibiting the generation of ROS and interfering with the release of inflammatory factors.

Mainly secreted by adipocytes, adiponectin is a kind of adipokines that can regulate metabolic diseases including obesity and diabetes mellitus.^16^ Studies have shown that adiponectin can exert its inhibitory effect on inflammation after cerebral haemorrhage by regulating mitochondrial function.^17^ Notably, adiponectin can accelerate wound healing via the upregulation of keratin gene transcripts, collagen organization and proliferation of cells in the basal layer of the epidermis.^18^ Besides, adiponectin can regulate psoriasiform skin inflammation by suppressing IL‐17 production of γδ‐T cells.^19^ However, excessive adiponectin may deliver serious adverse effects like reduced bone density and weight gain.^20^ As a newly developed agonist of adiponectin receptors, AdipoRon (AR) exerts similar positive effects like adiponectin with fewer adverse effects. It has been shown that AR improved insulin sensitivity and extended the lifespan of db/db mice, a rodent model of type II diabetes via promoting the phosphorylation of AMP‐activated protein kinase (AMPK).^21^ However, the effect of AR on cellular senescence and inflammation of the skin by affecting mitochondrial function remains unclear. Therefore, we aim to verify the effect of AR on the SASP and inflamm‐ageing of skin and ESCs, and provide a basis for clinical use in the future.

## 2 MATERIALS AND METHODS

### 2.1 Animals and treatments

1‐month (1M), 13‐month (13M) and 23‐month (23M) male C57BL/6 (B6) mice were purchased from the SPF (Beijing) Biotechnology Co., Ltd. We specified to receive mice with ‘good hair coats’ to avoid obvious dermatitis, fighting, scratching and inflamed animals. The tumour‐free mice were selected for experiments. All mice were housed in a specific pathogen‐free microisolator environment with constant temperature (23 °C), humidity (60%) and a 12‐h light/dark cycle at The Fourth Medical Center of PLA General Hospital. To detect the effect of age on inflammatory factors and apoptosis in the skin, AR (10, 20, 40 mg/kg) was dissolved in 0.5% sodium carboxymethyl cellulose solution and was provided to 23M mice in therapy group once daily via intragastric gavage for 4 weeks. 0.5% sodium carboxymethyl cellulose solution was provided to 1M, 13M and 23M mice in the control group once daily via intragastric gavage for 4 weeks. *N* = 8 for each group. Procedures were performed using IACUC‐approved protocols that adhere to NIH standards.

### 2.2 Wounding study

Punch biopsies were performed on anaesthetized mice for wounding experiments. For dorsal skin wounds, dorsal hairs were cut with clippers, and skin was swabbed with EtOH prior to wounding. Depilation was performed and 8‐mm biopsy punches were used to make full‐thickness wounds as described.^22^ The removed dorsal skin samples were used for immunofluorescence and Western blot as described below. After, mice were housed separately, and photos were taken at day (d) 0.5, d3, d5, d7 and d9 to compare wound healing rate. To detect the effect of AR on wound healing rate, AR (40 mg/kg) was dissolved in 0.5% sodium carboxymethyl cellulose solution and was provided to 24M mice once daily via intragastric gavage till d9. 0.5% sodium carboxymethyl cellulose solution was provided to the 2M, 14M and 24M mice in the control group once daily via intragastric gavage till d9. *N* = 8 for each group.

### 2.3 Immunofluorescence

Dorsal skin tissue was fixed in 4% paraformaldehyde (P0099, Beyotime), rinsed with phosphate buffered saline (PBS), permeabilized 10 min with 0.1% Triton X‐100 (HFH10, Invitrogen) in PBS and then blocked for 1 h in 2.5% normal goat serum (R37624, Invitrogen). Primary antibodies (and their dilutions) used were as follows: tumour necrosis factor‐α (TNF‐α) Rabbit antibodies (Ab) (11948, 1:200, Cell Signaling Technology), Interleukin‐6 (IL‐6) Rabbit Ab (ab208113, 1:200, Abcam) and IL‐1β Rabbit Ab (31202, 1:200, Cell Signaling Technology). Primary antibodies were diluted in 2.5% normal goat serum and incubated at 4 °C overnight. After washing with PBS, secondary antibodies, conjugated with Alexa488 (ab150073, 1:200, Abcam), Alexa594 (ab150064, 1:200, Abcam) or Alexa647 (ab150075, 1:200, Abcam), were added for 2 h at room temperature (RT). Slides were washed with PBS, counterstained with ProLong Diamond Antifade Mountant with 4′,6‐diamidino‐2‐phenylindole (DAPI) (P36962, Invitrogen). Images were acquired with a confocal microscope (SP8, Leica). The mean fluorescence intensity in five randomly chosen areas was calculated by ImageJ. The mean fluorescence intensity of DAPI was used as a reference.

### 2.4 Western blot

For tissue samples, after the subcutaneous fat was removed with a scalpel, the dorsal skin samples of mice were immediately frozen in liquid nitrogen. Frozen tissues were homogenized using a tissue grinder and collected in RIPA Lysis and Extraction Buffer (89901, Thermo Fisher) with protease inhibitors (HY‐B0496, MedChemExpress) and phosphatase inhibitors (HY‐K0021, HY‐K0022, MedChemExpress). For cell samples, the treated cells were washed with pre‐chilled PBS and then collected in lysis buffer. Tissue and cell debris were removed by centrifugation. Protein concentrations were measured using a bicinchoninic acid (BCA) protein assay kit (23227, Thermo Fisher). The lysate was boiled for 5 min in 5× Sodium dodecyl sulphate (SDS) loading buffer (P1040, Solarbio) containing 5% β‐mercaptoethanol. 60 μg samples were then subjected to SDS‐polyacrylamide gel electrophoresis (PAGE) on 10% Hepes‐Tris Precast‐Gel (PG01010‐S, Solarbio) and transferred to polyvinylidene difluoride (PVDF) membranes (IPVH00010, Millipore). After blocked with 5% non‐fat milk in Tris Buffered saline Tween (TBST) (pH 7.6) for 1 h, the membranes were incubated with corresponding primary antibodies overnight at 4 °C. Then, membranes were incubated with the appropriate horseradish peroxidase (HRP)‐conjugated secondary antibodies at RT for 2 h. The antibody incubations were then followed by three times 5‐min TBST washes. The protein bands were detected with ChemiDoc XRS chemiluminescence imaging system (Bio‐Rad) and quantified by Image Lab, an image acquisition and analysis software (Bio‐Rad). All antibodies used were as follows: TNF‐α Rabbit Ab (11948, 1:1000, Cell Signaling Technology), IL‐6 Rabbit Ab (ab208113, 1:1000, Abcam), IL‐1β Rabbit Ab (31202, 1:1000, Cell Signaling Technology), GAPDH Rabbit Ab (5174, 1:1000, Cell Signaling Technology), Cleaved Caspase‐9 Rabbit Ab (9509, 1:1000, Cell Signaling Technology), Cleaved Caspase‐3 Rabbit Ab (9661, 1:1000, Cell Signaling Technology), Bax Rabbit Ab (14796, 1:1000, Cell Signaling Technology), Bcl2 Rabbit Ab (3498, 1:1000, Cell Signaling Technology), dynamin‐related protein 1 (Drp1) Rabbit Ab (8570, 1:1000, Cell Signaling Technology), COX IV Rabbit Ab (4850, 1:1000, Cell Signaling Technology), Cytochrome C Rabbit Ab (ab133504, 1:5000, Abcam), Phospho‐Drp1 (Ser637) Rabbit Ab (ab193216, 1:800, Abcam), Phospho‐Drp1 (Ser616) Rabbit Ab (4494, 1:1000, Cell Signaling Technology), AMPKα Rabbit Ab (2532, 1:1000, Cell Signaling Technology), Phospho‐AMPKα (Thr172) Rabbit Ab (50081, 1:1000, Cell Signaling Technology), β Actin Mouse Ab (3700, 1:1000, Cell Signaling Technology), HRP‐conjugated Goat Anti‐Rabbit IgG(H+L) (SA00001‐2, 1:1000, Proteintech), HRP‐conjugated Goat Anti‐Mouse IgG(H+L) (SA00001‐1, 1:1000, Proteintech).

### 2.5 TUNEL staining

Briefly, the in situ cell death detection kit (C1089, Beyotime) was used to measure apoptosis in the dorsal skin of different ages of mice according to the manufacturer's instructions. The slices were treated with 20 μg/ml DNase‐free proteinase K (AM2542, Invitrogen) at 37 °C for 30 min and washed with PBS for 3 times. Then, the slices were dyed using the TUNEL reaction solution prepared in a humid dark box for 1 h at 37 °C. After washing with PBS, the tissues were dyed using ProLong Diamond Antifade Mountant with DAPI (P36962, Invitrogen). Images were acquired with a confocal microscope (SP8, Leica). The apoptotic index was counted and calculated as an average of five random visual fields by the ratio of TUNEL‐positive cells to DAPI‐positive cells.

### 2.6 Cell culture and treatments

The foreskin samples used in this study were donated by healthy adults aged 20–30 years who underwent surgical circumcision, and all donors were of the Asian race. Fresh foreskins were obtained with the donors’ consent and the approval of the Ethics Committee of The Fourth Medical Center of Chinese PLA General Hospital, Beijing, China. The foreskin tissue was surgically removed using aseptic techniques. The collected samples were kept in sterile physiological saline and transported at 2–8 °C to the laboratory for processing. To obtain the ESCs, subcutaneous fat was removed from skin samples with a scalpel, and skins were placed dermis side down in 2.4 U/ml Dispase II (17105041, Gibco) for 1 h. Single‐cell suspensions were obtained by scraping the skin to remove the epidermis from the dermis. Cells were then filtered through 70 mm, followed by 40‐mm strainers and plated on Collagen IV–coated 6‐well tissue culture dishes to establish primary cell lines as described.^23^ Independent clones were cultured and passaged in Epilife culture medium (MEPI500CA, Gibco) with Human Keratinocyte Growth Supplement (HKGS) (S0015, Gibco). The 2nd and 3rd passages were used in the experiments. For experiments, cells were cultured in EpiLife culture medium with or without Lipopolysaccharide (LPS) (1 mg/L), AR (40 μM), mitochondrial division inhibitor 1 (Mdivi‐1) (10 μM), or Dorsomorphin (Dor) (20 μM) for indicated hours. LPS was used to simulate the inflammatory environment in aged skin. AR is a new type of oral agonist acting selectively on adiponectin receptors 1 and 2. Mdivi‐1 is a selective and cell‐penetrating mitochondrial division inhibitor that inhibits Drp1. Dor is an effective, reversible, selective AMPK inhibitor.

### 2.7 Transmission electron microscopy

Skin samples were obtained from 2M, 14M and 24M mice with corresponding treatments. After fixation in 4% glutaraldehyde overnight, the sections were fixed in 1% osmium‐tetroxide for 1 h, dehydrated using a graded ethanol immersion series and then embedded in resin. The tissue section was cut into 60–80 nm sections using an ultramicrotome (UC7, Leica). The ultrathin section was fixed on a 200‐slot grid coated with pioloform support film and observed using an electron microscope (HT7700, Hitachi).

### 2.8 Mitochondrial isolation and detection

To isolate the mitochondria and cytoplasm, ESCs were suspended by PBS after gathering from each culture plate. The cell suspension was centrifuged at 600 *g* for 5 min at 4 °C. Then, the cell pellets were suspended by mitochondrial separation reagent containing 1% phenylmethylsulfonyl fluoride (PMSF) for 15 min at 4 °C. The cell suspension was homogenized by glass homogenizer properly. Afterwards, the mixture was centrifuged at 600 *g* for 10 min at 4 °C, and then, the supernatant was centrifuged at 12,000 *g* for 10 min at 4 °C. The sediment was the mitochondria isolated from ESCs. The supernatant was the cytoplasmic proteins. To collect mitochondrial proteins, the mitochondrial sediment was incubated in lysis buffer containing 1% PMSF for 30 min to harvest the mitochondrial proteins for Western blot analysis. To collect cytoplasmic proteins, the supernatant was centrifuged at 12,000 *g* for 10 min at 4 °C and the supernatant now was the cytoplasmic protein that removed the mitochondria. The mitochondrial separation reagent, lysis buffer and PMSF were provided by the Cell mitochondrial separation Kit (C3601, Beyotime). For the extraction of mitochondria from skin tissues, a 50‐mg sample was cut, washed with pre‐cooled PBS and extracted using a tissue mitochondrial isolation kit (C3606, Beyotime), following the above steps under the guidance of the instructions.

To detect the morphological changes of the mitochondria, the treated ESCs were transduced with CellLight Mito‐GFP (C10600, Invitrogen) with a particle‐per‐cell value of 20. Thus, 20 μl BacMam 2.0 reagent was added to 10^5^ cells/well in one well of a 96‐well plate following the manufacturer's protocol. The transduction was performed in Epilife culture medium with HKGS for 24 h. After washed with pre‐warmed PBS for 3 times, the cells were given indicated treatments for 24 h, and then, the morphological changes of the mitochondria were observed under a confocal microscope (SP8, Leica). Following the protocol, the ImageJ plug‐in MiNA was used to detect and count mitochondrial footprint and mean branch lengths to compare the fragmentation of mitochondria.^24^, ^25^

To detect the changes in mitochondrial ROS generation, the treated ESCs were stained in 5 nM MitoSOX Red Mitochondrial Superoxide Indicator solution (M36008, Invitrogen) for 30 min at 37 °C. The fluorescence images were captured via a confocal microscope (SP8, Leica) under 510 nm excitation wavelength and 580 nm emission wavelength. Quantitation of mean fluorescence intensity by ImageJ was used to compare the ROS changes of the mitochondria.

To detect the changes in mitochondrial membrane potential (MMP), the treated ESCs were incubated in JC‐1 solution (T3168, Invitrogen) at a concentration of 10 mg/L for 20 min at 37 °C and then washed 3 times with PBS to remove excess JC‐1 solution. Then, the stained samples images were immediately captured under a confocal microscope (SP8, Leica). Lastly, the relative MMP was analysed with the ImageJ software. The relative MMP was calculated as a ratio of the mean fluorescence intensity of red fluorescence (excitation wavelength, 525 nm; emission wavelength, 590 nm) to green fluorescence (excitation wavelength, 490 nm; emission wavelength, 530 nm).

To detect the changes in mitochondrial ATP synthesis function, the treated ESCs were lysed and tested with ATP Determination Kit (A22066, Invitrogen), following the manufacturer's instructions.

### 2.9 Cell apoptosis

To detect cell apoptosis, preparation of ESCs and staining protocols were done with FITC Annexin‐V Apoptosis Detection Kit I (556547, BD) per manufacturer's instructions.

### 2.10 Sulforhodamine B assay

The effect of LPS and AR on cell proliferation was investigated using the sulforhodamine B (SRB) assay. In brief, 10^4^ cells were loaded into sterile 96‐well plates containing 100 μL of Epilife culture medium with HKGS and incubated 24 h for cell adherence. The wells were then washed with PBS and replenished with Epilife culture medium with different drugs for 12, 24 and 36 h. At indicated time points after treatment, cells were fixed with pre‐cooled trichloroacetic acid and incubated at 4 °C for 1 h. The plates were then washed 3 times with deionized water and stained with SRB dye (0.4% dissolved in 1% acetic acid) for 25 min at RT. Wells were then washed 3 times with 1% acetic acid to remove any unbound dye and dried in the air at RT. 100 μL 10 mM Tris base was added to each well to solubilize bound dye and gently mixed to obtain a homogenous solution. Finally, absorbance was measured at 510 nm by a microplate reader (Synergy2, BioTek) per the manufacturer's instructions.

### 2.11 Cell migration assays

The scratch‐wound healing assay was performed essentially as described.^22^ In brief, ESCs were plated on 6‐well tissue culture dishes. After cells reached confluency, wounds were created by the manual scraping of the cell monolayer with a pipette tip. The dishes were then washed with PBS, replenished with Epilife culture medium with different drugs and photographed with a phase‐contrast microscope. Afterwards, dishes were placed in the tissue culture incubator, and the matched wound regions were photographed 12, 24 and 36 h after wounding. The ImageJ software was used to compare the relative wound healing rate.

### 2.12 Cell mitochondria stress test

Oxygen consumption rates (OCR) and ATP production were measured using the Seahorse Bioscience XF24 Extracellular Flux Analyzer according to the protocol.^26^ Cells were seeded in XFe24 cell culture plates and incubated at 37 °C in incubator with 5% CO_2_ for cell attachment. For the experiment, cells were replenished with corresponding medium for 24 h. Before the experiment, cells were washed and changed to seahorse assay medium (supplement with 10 mM Glucose, 1 mM Pyruvate, 2 mM Glutamine and adjusted to pH 7.4), and then incubated in non‐CO_2_ incubator for 1 h. After measuring the basal OCR level, the ATP synthase inhibitor Oligomycin (1.5 μM), the uncoupler carbonyl cyanide 4‐(trifluoromethoxy) phenylhydrazone (FCCP, 0.5 μM) and the mitochondrial electron transport chain inhibitors Antimycin/Rotenone (0.5 μM) were sequentially injected into the cell chamber according to Seahorse standard protocols. The cells in each well were lysed by RIPA to extract the protein, and the protein concentration was measured and used to normalize the data for eliminating deviations caused by differences in the number of cells between wells. The OCR was expressed as pmol/min/mg/ml. The basal OCR level was calculated before the injection of Oligomycin. The maximal OCR level was calculated between the injection of FCCP and Antimycin/Rotenone.

### 2.13 Quantification and statistical analysis

The statistical analysis was performed by two‐tailed unpaired Student's *t* test, one‐way or two‐way analysis of variance (ANOVA) and presented as the mean ± SEM using the Prism software (GraphPad). For all statistical tests, the 0.05 level of confidence was accepted as a significant difference.

## 3 RESULTS

### 3.1 AdipoRon diminished the enhanced inflammation and apoptosis levels during skin ageing

**FIGURE 1:**
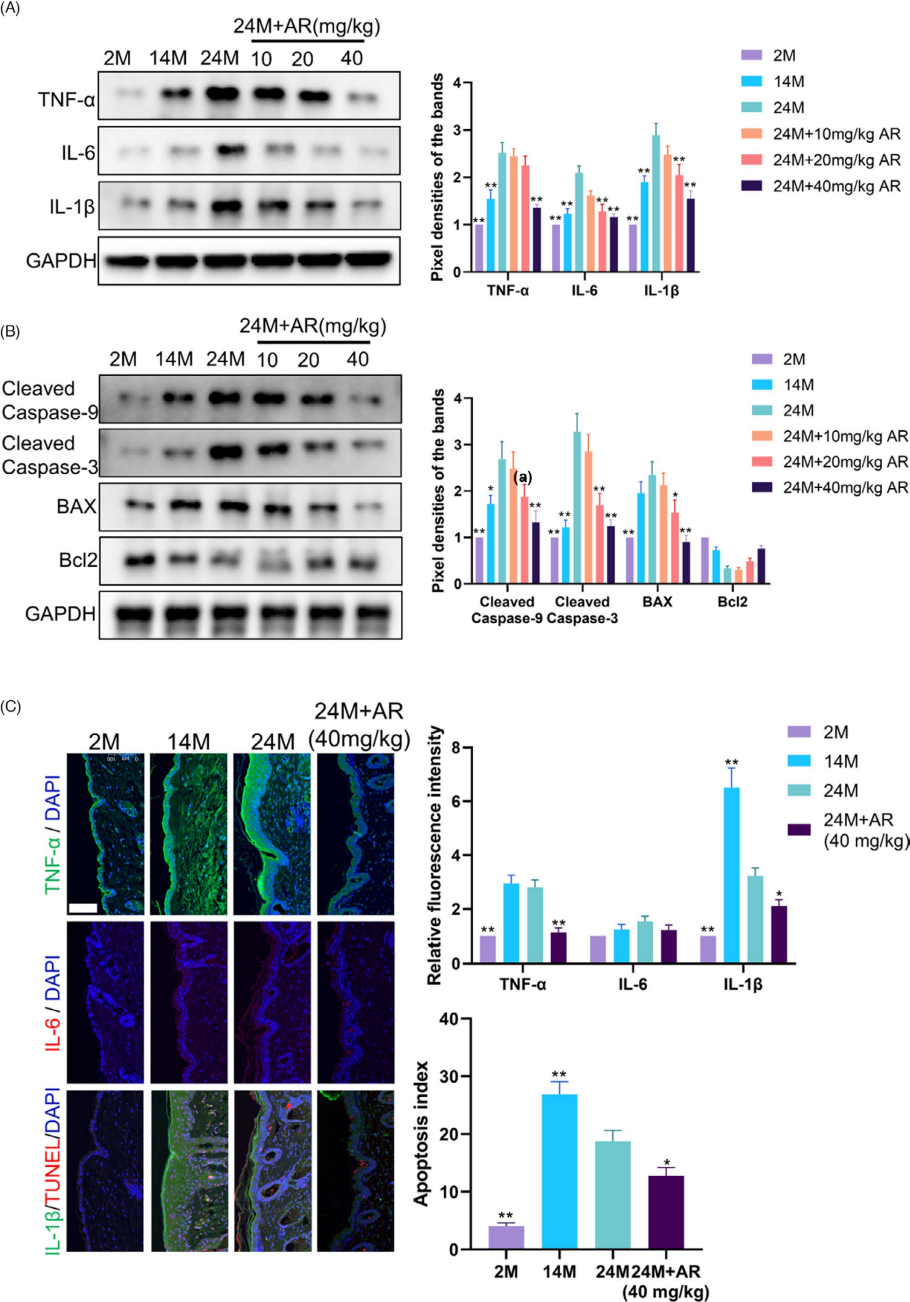
AdipoRon Diminished the Enhanced Inflammation and Apoptosis Levels during Skin Ageing. (A) Western blot was used to measure the expression level of TNF‐α, IL‐6 and IL‐1β in skin samples obtained from different groups. *N* = 8. ^*^ *p* < 0.05 and ^**^ *p* < 0.01 versus 24M group. (B) Western blot was used to measure the expression level of cleaved caspase‐9, Cleaved Caspase‐3, BAX, Bcl2 in skin samples obtained from different groups. *N* = 8. ^*^ *p* < 0.05 and ^**^ *p* < 0.01 versus 24M group. (C) Immunofluorescence images of skin samples labelled with antibodies (Abs) against TNF‐α, IL‐6 and IL‐1β [secondary Abs are colour‐coded as shown]. TUNEL staining is used to mark apoptotic cells. Sections were co‐stained with DAPI (blue) to visualize nuclei. *N* = 8. Scale bar, 100 μm. ^*^ *p* < 0.05 and ^**^ *p* < 0.01 versus 24M group

Western blot revealed that compared with 2M mice, 14M mice and 24M mice exhibited higher inflammatory factors TNF‐α, IL‐6 and IL‐1β in the skin, whereas AR treatment dose‐dependently improved the inflammatory response (Figure 1A). Immunofluorescence revealed that increased inflammatory factors with age were mainly distributed in the epidermis and decreased to a certain extent with AR (40 mg/kg) treatment (Figure 1C). Not surprisingly, Western blot showed that AR dose‐dependently inhibited the expression of apoptosis‐related proteins cleaved caspase‐9, cleaved caspase‐3 and Bax that increased with age (Figure 1B). Meanwhile, AR gave rise to elevated Bcl2 which declined with age (Figure 1B). Correspondingly, TUNEL staining showed that there were few apoptotic cells in 2M skin (Figure 1C). The apoptotic cells that increased with age were mainly distributed in the epidermis and hair follicles, which was alleviated after AR (40 mg/kg) treatment (Figure 1C).

### 3.2 AdipoRon reversed the changes in mitochondrial morphology and function during ageing and inhibited the mitochondrial translocation of Drp1

**FIGURE 2:**
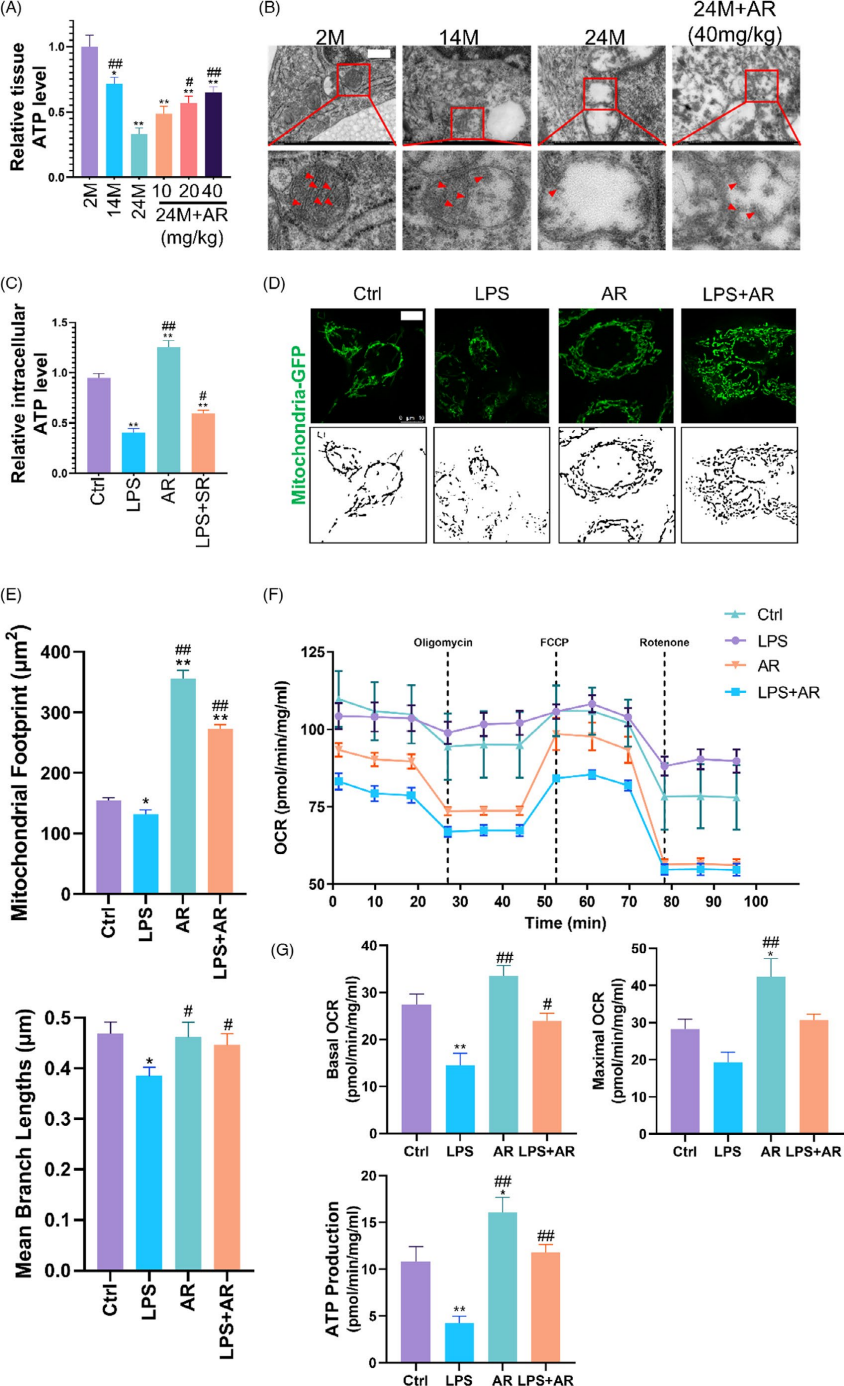
AdipoRon Reversed the Changes in Mitochondrial Morphology and Function during Skin Ageing. (A) The ATP level was detected to reflect the changes in mitochondrial function in skin samples obtained from different groups. *N* = 8. ^*^ *p* < 0.05 and ^**^ *p* < 0.01 versus 2M group. ^#^ *p* < 0.05 and ^##^ *p* < 0.01 versus 24M group. (B) Electron microscopy was performed to reflect the ultrastructural changes in ESCs of each group of mice. The box area below each photomicrograph shows an enlarged version of the red square. The red arrows indicate mitochondrial cristae. *N* = 8. Scale bar, 500 nm. (C) The ATP level was detected to reflect the changes in mitochondrial function in ESCs with different treatments. *N* = 3. ^*^ *p* < 0.05 and ^**^ *p* < 0.01 versus control group. ^#^ *p* < 0.05 and ^##^ *p* < 0.01 versus LPS group. (D) and (E) Labelled by CellLight Mito‐GFP, the mitochondrial morphology changes of ESCs in different treatment groups were observed. Skeletonized images were listed below to show the integrity of mitochondria. Scale bar, 10 μm. *N* = 3. ^*^ *p* < 0.05 and ^**^ *p* < 0.01 versus control group. ^#^ *p* < 0.05 and ^##^ *p* < 0.01 versus LPS group. (F) and (G) The mitochondrial stress test of the Seahorse assay was used to reflect changes in the mitochondrial respiratory function of ESCs. *N* = 5. ^*^ *p* < 0.05 and ^**^ *p* < 0.01 versus control group. ^#^ *p* < 0.05 and ^##^ *p* < 0.01 versus LPS group

As the main energy source for cell metabolism, ATP is mainly synthesized in mitochondria, which are the main energy supply organs in cells. The content of ATP can reflect the mitochondrial function. We found that the content of ATP in the skin decreased with age, while the treatment of 20 and 40 mg/kg AR triggered an elevated level of ATP in the skin of 24M mice (*p *< 0.05) (Figure 2A). This indicated that AR ameliorated the function of mitochondria damaged in aged skin.

Transmission electron microscopy was used to observe the ultrastructure changes of mitochondria in the skin of mice of different ages (Figure 2B). There were long tubular mitochondrial structures, prominent cristae and some small spherical mitochondrial structures in the 2M skin. However, the 24M skin was characterized by swelling of mitochondria, loss of cristae and mitochondria collapse. These morphological changes in 24M mice were partly reversed by AR treatment. This showed that AR had a certain protective effect on mitochondrial morphology affected by age.

LPS was used to incubate ESCs in in vitro experiments to simulate the inflamm‐ageing of skin as described.^27^ Compared with the control group, AR significantly increased the intracellular ATP content (*p* < 0.01), and ESCs synthesized a lower level of ATP after incubated with LPS (*p* < 0.01), which was reversed by AR treatment (*p* < 0.05) (Figure 2C). CellLight Mito‐GFP was used to evaluate the influence of AR on the mitochondrial morphology of ESCs under inflammation (Figure 2D,E). Compared with the control group, the mitochondria in the AR group showed better integrity with higher mitochondrial footprints (*p* < 0.01), but the mitochondrial fragments in the LPS group increased significantly with lower mitochondrial footprints and mean branch lengths (*p* < 0.05). Compared with the LPS group, AR treatment partially reversed the morphological damage of the mitochondria and restored their integrity in the presence of LPS, reflected as better mitochondrial footprints and mean branch lengths (*p* < 0.05) (Figure 2D,E). These data indicated that AR played a certain protective effect against the mitochondrial damage induced by inflammatory factors in ESCs.

To reveal the biological process behind the mitochondrial respiration in cells dynamically, a mitochondria stress test was performed to monitor the changes in OCR and ATP production (Figure 2F,G). Compared with the control group, exposure to LPS for 24 h significantly reduced the basal OCR (*p* < 0.01) and ATP production (*p* < 0.01) of ESCs; AR promoted the maximum OCR (*p* < 0.05) and ATP production (*p* < 0.05) on ESCs rather than the basal OCR (*p* > 0.05). Compared with the LPS group, AR treatment improved the basal OCR (*p* < 0.05) and ATP production (*p* < 0.01) of ESCs in the presence of LPS.

**FIGURE 3:**
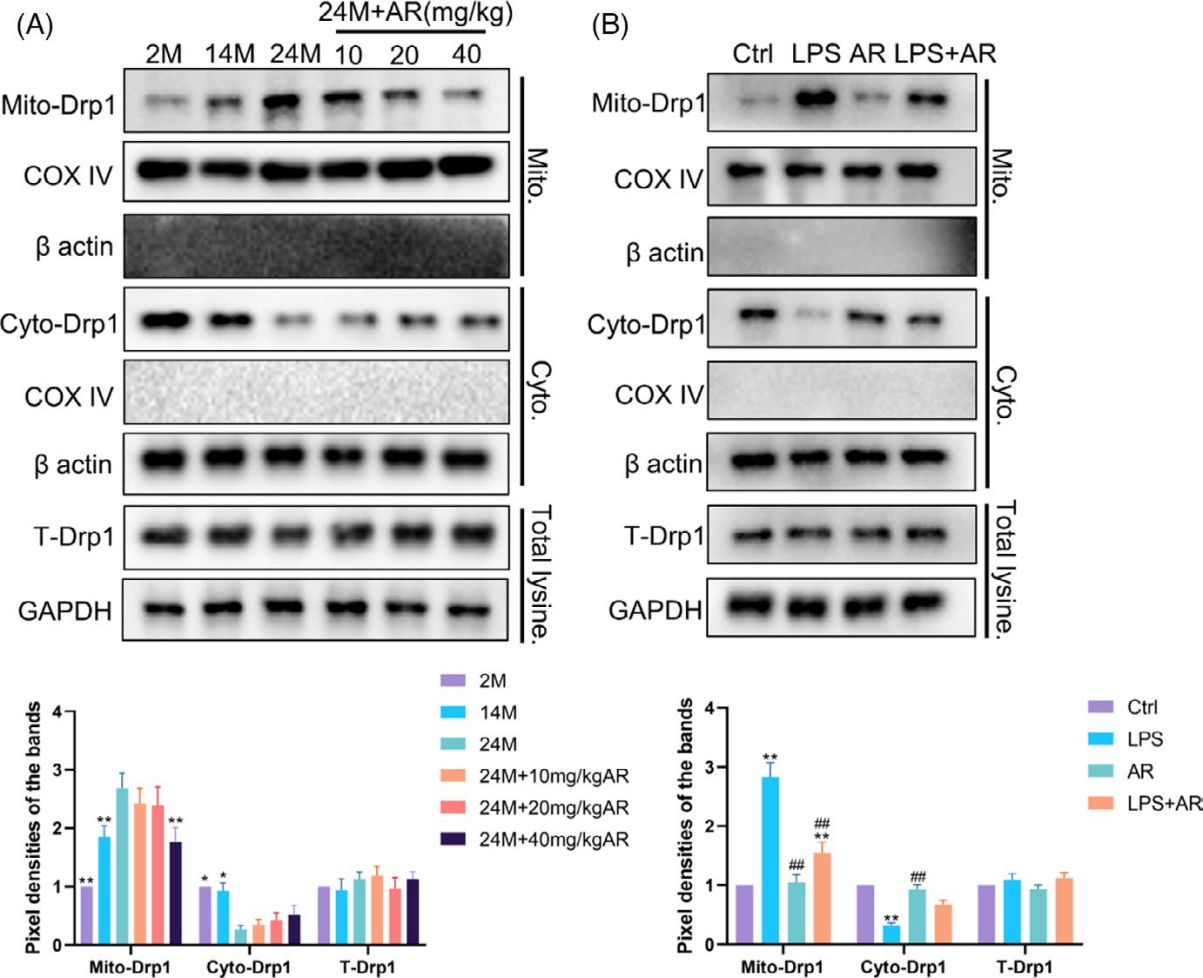
AdipoRon Suppressed the Mitochondrial Translocation of Drp1 in Aged Skin or ESCs under inflammation. (A) Western blot was used to measure the distribution of Drp1 in mitochondria and cytoplasm from samples obtained from mice in different groups. *N* = 8. ^*^ *p* < 0.05 and ^**^ *p* < 0.01 versus 24M group. (B) Western blot was used to measure the distribution of Drp1 in mitochondria and cytoplasm in ESCs with different treatments. *N* = 3. ^*^ *p* < 0.05 and ^**^ *p* < 0.01 versus control group. ^#^ *p* < 0.05 and ^##^ *p* < 0.01 versus LPS group

Acting as a key process of mitochondrial fission, mitochondrial translocation of Drp1 promotes mitochondrial fragmentation in many cases.^28^ Here, we found that the total Drp1 protein level in the skin of mice of different ages did not change significantly (Figure 3A). However, compared with the 2M group, Drp1 decreased in the cytoplasm of 24M mice skin but increased in mitochondria. This trend was reversed by AR treatment (Figure 3A). Accordingly, LPS triggered an obvious trend of the mitochondrial translocation of Drp1, while AR reversed this situation in ESCs in the presence of LPS (Figure 3B). These data indicated that inflammation could promote the mitochondrial translocation of Drp1. AR could suppress the excessive mitochondrial division caused by mitochondrial translocation of Drp1 during the inflamm‐ageing of skin.

### 3.3 AdipoRon inhibited mitochondrial oxidative stress and inflammation mediated by the mitochondrial translocation of Drp1

**FIGURE 4:**
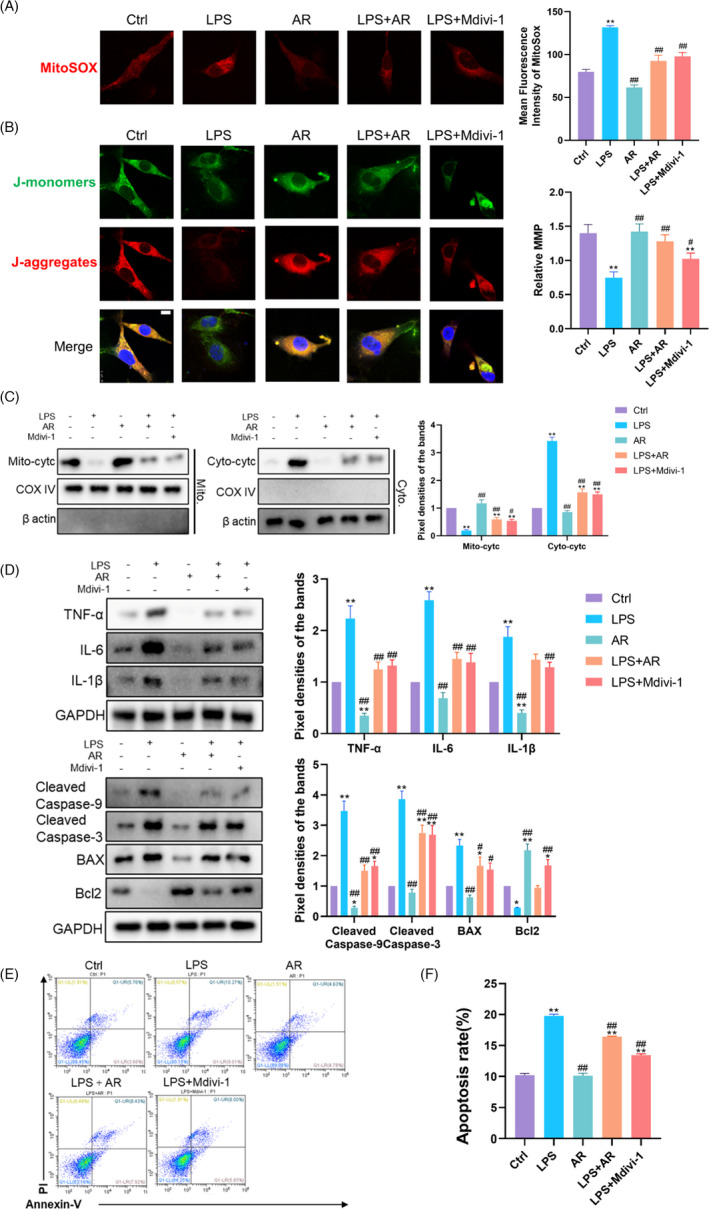
AdipoRon Inhibited Mitochondrial Oxidative Stress and Inflammation Mediated by the Mitochondrial Translocation of Drp1. (A) MitoSOX was used to reflect the ROS level in mitochondria in ESCs with different treatment. *N* = 3. The mean fluorescence intensity was used for statistical analysis. ^*^ *p* < 0.05 and ^**^ *p* < 0.01 versus Ctrl, ^#^ *p* < 0.05 and ^##^ *p* < 0.01 versus LPS. (B) The mitochondrial membrane potential of cells was measured via JC‐1 staining. *N* = 3. Relative MMP was quantified using ImageJ software. ^*^ *p* < 0.05 and ^**^ *p* < 0.01 versus Ctrl, ^#^ *p* < 0.05 and ^##^ *p* < 0.01 versus LPS. (C) Western Blot was used to measure the distribution of Cytochrome C in mitochondria and cytoplasm in ESCs with different treatments. *N* = 3. ^*^ *p* < 0.05 and ^**^ *p* < 0.01 versus Ctrl, ^#^ *p* < 0.05 and ^##^ *p* < 0.01 versus LPS. (D) Western blot was used to measure the inflammation and apoptosis levels in ESCs with different treatments. *N* = 3. ^*^ *p* < 0.05 and ^**^ *p* < 0.01 versus Ctrl, ^#^ *p* < 0.05 and ^##^ *p* < 0.01 versus LPS. (E) and (F) Flow cytometry was further used to quantify apoptosis in cells. *N* = 3. ^*^ *p* < 0.05 and ^**^ *p* < 0.01 versus Ctrl, ^#^ *p* < 0.05 and ^##^ *p* < 0.01 versus LPS

The selective Drp1 inhibitor Mdivi‐1 was used as a positive control to evaluate the effects of AR on the mitochondria of ESCs by regulating Drp1. Reflected by the mean fluorescence intensity of MitoSOX, mitochondrial ROS had a tendency to decrease after AR treatment with no statistical difference (*p* > 0.05), while LPS caused a significant increase in mitochondrial ROS (*p* < 0.01) (Figure 4A). Like Mdivi‐1, AR reversed LPS‐induced mitochondrial ROS generation in ESCs (*p* < 0.01) (Figure 4A). MMP was measured by JC‐1 staining (Figure 4B). Compared with the control group, AR alone had no statistical effect on MMP (*p* > 0.05), while LPS caused a significant decrease in MMP (*p* < 0.01). Yet, AR and Mdivi‐1 reversed the decreased MMP in the presence of LPS in ESCs statistically (*p* < 0.05). Cytochrome C (Cytc) is a carrier of electrons in the mitochondrial respiratory chain, and its transfer from mitochondria to cytoplasm is known as mitochondrial outer membrane permeabilization (MOMP). Besides eliciting caspase activation, MOMP engages various pro‐inflammatory signalling functions. The use of AR alone had no significant effect on the release of Cytc from mitochondria to cytoplasm. LPS induced the release of Cytc from mitochondria (*p* < 0.01), while AR and Mdivi‐1 partially reversed this transfer in ESCs (*p* < 0.05) (Figure 4C). Accordingly, AR or Mdivi‐1 also blocked the enhanced expression of TNF‐α, IL‐6 and IL‐1β induced by LPS (Figure 4D) and resulted in a lower level of apoptosis in ESCs (Figure 4D–F). These results indicated that AR could relieve ESCs from oxidative damage and inflammation, which dependent on the inhibition of Drp1‐mediated mitochondrial excessive fission.

### 3.4 AdipoRon inhibited Drp1‐mediated mitochondrial excessive division by phosphorylating AMPK

**FIGURE 5:**
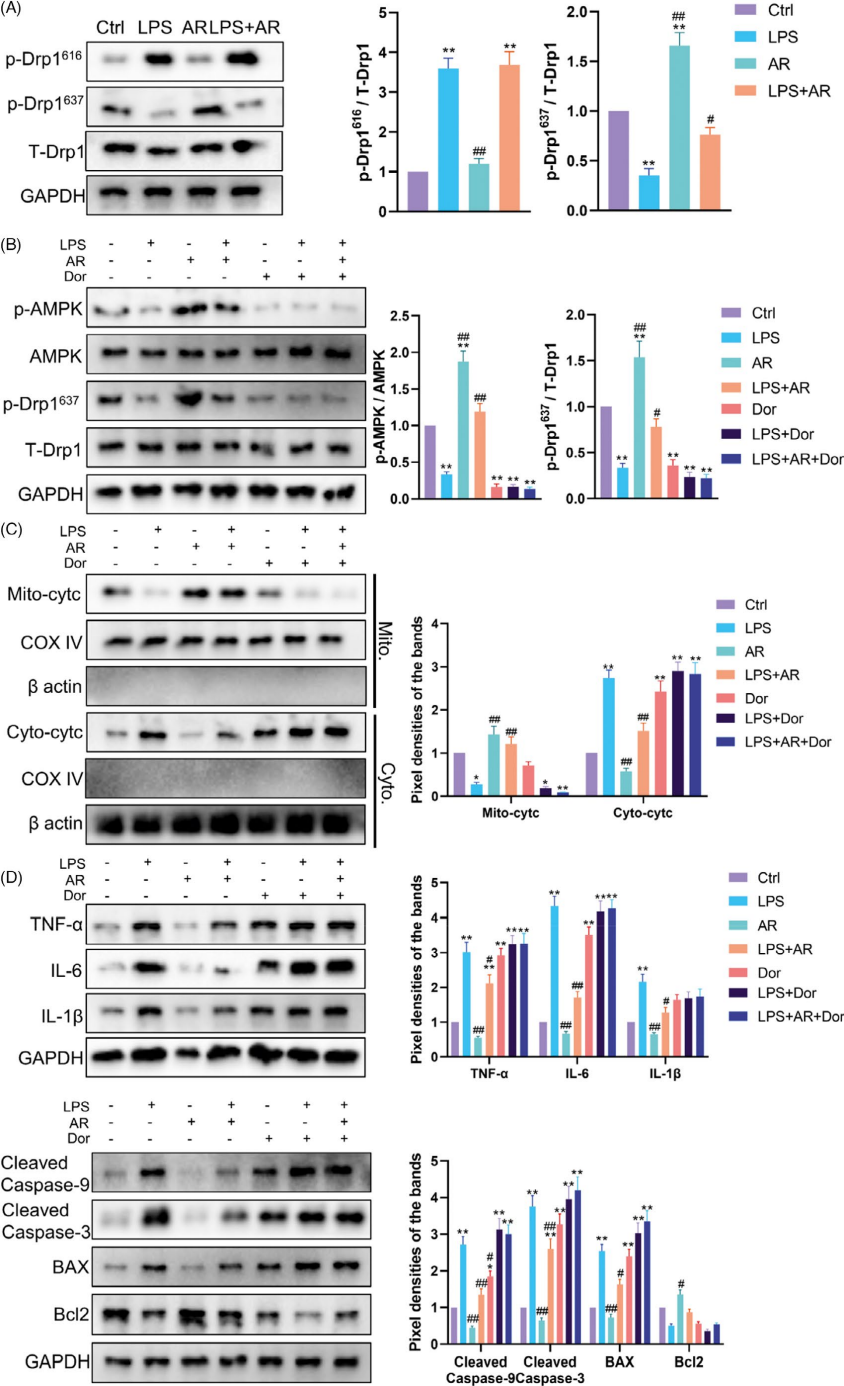
AdipoRon Inhibited Drp1‐mediated Mitochondrial Excessive Division by Phosphorylating AMPK. (A) Western blot was used to measure the phosphorylation level of Drp1 at serine 616 and 637 in ESCs with different treatments. *N* = 3. ^*^ *p* < 0.05 and ^**^ *p* < 0.01 versus Ctrl, ^#^ *p* < 0.05 and ^##^ *p* < 0.01 versus LPS. (B) Western blot was used to measure the phosphorylation level of AMPKα and Drp1 in ESCs with different treatments. *N* = 3. ^*^ *p* < 0.05 and ^**^ *p* < 0.01 versus Ctrl, ^#^ *p* < 0.05 and ^##^ *p* < 0.01 versus LPS. (C) Western blot was used to measure the distribution of Cytochrome C in mitochondria and cytoplasm in ESCs with different treatments. *N* = 3. ^*^ *p* < 0.05 and ^**^ *p* < 0.01 versus Ctrl, ^#^ *p* < 0.05 and ^##^ *p* < 0.01 versus LPS. (D) Western blot was used to measure the inflammation and apoptosis levels in ESCs with different treatments. *N* = 3. ^*^ *p* < 0.05 and ^**^ *p* < 0.01 versus Ctrl, ^#^ *p* < 0.05 and ^##^ *p* < 0.01 versus LPS

**FIGURE 6:**
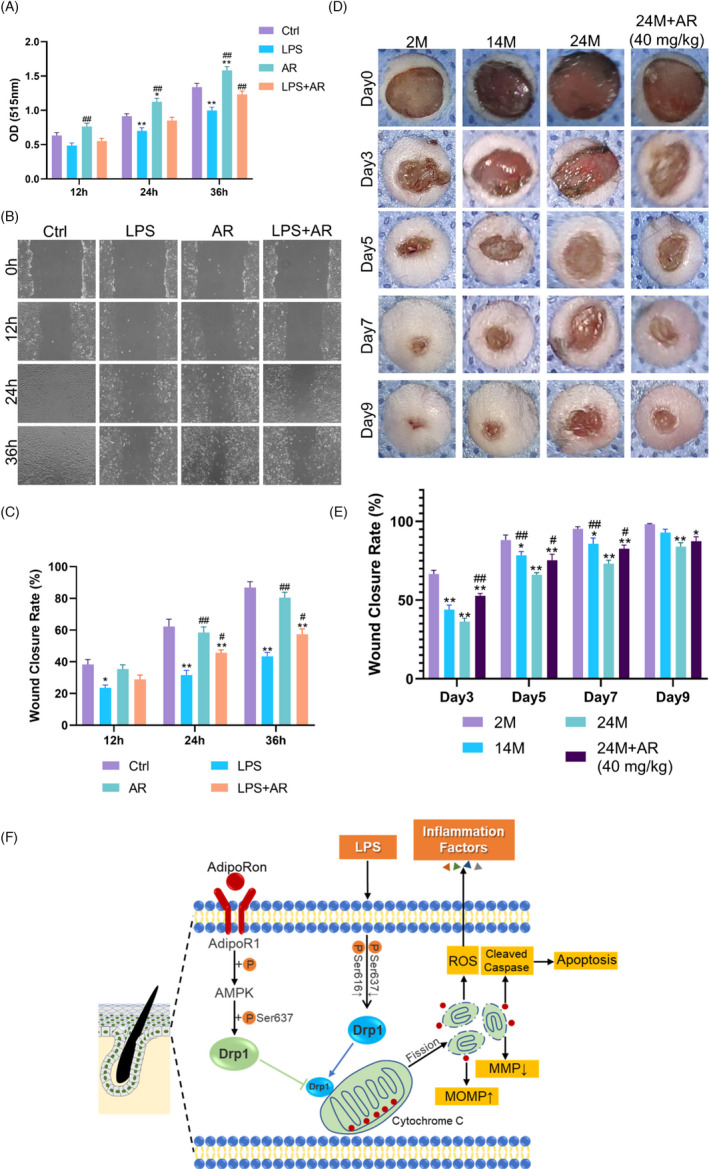
AdipoRon Promoted Wound Healing in Aged Skin through Ameliorating Cell Proliferation and Migration of ESCs under Inflammation. (A) The proliferation effect of AdipoRon on ESCs is compared by the absorbance at 510 nm in the SRB assay. *N* = 3. ^*^ *p* < 0.05 and ^**^ *p* < 0.01 versus Ctrl, ^#^ *p* < 0.05 and ^##^ *p* < 0.01 versus LPS. (B) and (C) Confluent monolayers of cells were randomly divided into four groups and subjected to in vitro scratch‐wound assays. Migration into ~600 μm scratch wounds of cells was measured by phase‐contrast images of the site taken at indicated hours after scratch wounding. Quantifications of wound closure are shown in the graph below. *N* = 3. ^*^ *p* < 0.05 and ^**^ *p* < 0.01 versus Ctrl, ^#^ *p* < 0.05 and ^##^ *p* < 0.01 versus LPS. (D) and (E) We generated 8 mm full‐thickness cutaneous wounds on the backs of animals. The blue fenestrated sheet has a diameter of 10 mm and is used as a reference. (D) Gross view of wounds with different treatments post‐wounding. *N* = 8. (E) The wound healing rates among the groups were quantified by using digital images evaluated with ImageJ software. ^*^ *p* < 0.05 and ^**^ *p* < 0.01 versus 2M, ^#^ *p* < 0.05 and ^##^ *p* < 0.01 versus 24M. *N* = 8. (F) A working model summarizing the role of AdipoRon in regulating Drp1‐mediated mitochondrial damage under inflammation. AdipoRon could effectively activate the AMPK pathway and reverse the down‐regulation of the phosphorylation level of Drp1 at serine 637 under LPS, thus attenuate excessive mitochondrial translocation of Drp1. The repression of the mitochondrial fragments by AdipoRon protected the morphology and function of mitochondria, thus contributing to an alleviated ROS, inflammation and apoptosis levels

It is reported that the phosphorylation of Drp1 at serine 616 (S616) accelerates its mitochondrial translocation, while the phosphorylation of Drp1 at serine 637 (S637) hinders this process. To determine the underlying mechanism of AR regulating the distribution of Drp1, we evaluated the effect of AR on Drp1 phosphorylation in ESCs (Figure 5A). Compared with the control group, AR increased the phosphorylation of Drp1 at S637 (*p* < 0.01) with little influence at S616 (*p* > 0.05), while LPS triggered the phosphorylation of Drp1 at S616 (*p* < 0.01) with a decreased phosphorylation at S637 (*p* < 0.01). Compared with the LPS group, AR treatment increased the phosphorylation of Drp1 at S637 in the presence of LPS (*p* < 0.05). This suggested that AR regulated the distribution of Drp1 mainly via the phosphorylation of Drp1 at S637. As the classic downstream of AR/AdipoR1, AMPK has been proven to phosphorylate the S637 of Drp1. As a selective AMPK inhibitor, Dor is used to explore whether the phosphorylation of Drp1 at S637 by AR depends on AMPK activation. Western blot showed that Dor alone inhibited the phosphorylation of AMPK and Drp1 with Cytc partially released from the mitochondria to the cytoplasm (Figure 5B,C). Meanwhile, Dor inhibited the phosphorylation of AMPK, which could be phosphorylated by AR. Drp1's phosphorylation at S637 by AR was abolished in the presence of Dor in ESCs (Figure 5B). The protective effect of AR on LPS‐induced translocation of Cytc from mitochondria to cytoplasm was also blocked by Dor (Figure 5C). Subsequently, the inhibitory effect of AR on the TNF‐α, IL‐6 and IL‐1β induced by LPS in ESCs was also attenuated by Dor (Figure 5D). The same trend was seen in apoptosis‐related proteins (Figure 5D). These data indicated that AR’s inhibition on Drp1‐mediated mitochondrial excessive division depended on the phosphorylation of Drp1 at S637 by activating AMPK, which in turn reduced the level of inflammation factors and apoptosis. Figure 6F was used to summarize the possible mechanism of AR regulating the shape and function of the mitochondria of ESCs affected by SASP through Drp1.

### 3.5 AdipoRon promoted wound healing in aged skin through ameliorating cell proliferation and migration

The SRB assay demonstrated that AR alone could promote the proliferation of ESCs at 24 h (*p* < 0.05) and 36 h (*p* < 0.01). In addition, AR reversed the inhibitory effect of LPS on cell proliferation after ESCs were incubated with LPS for 36 h (*p* < 0.01) (Figure 6A). In the scratch‐wound healing assay, AR alone showed little influence on cell migration (*p* > 0.05), while LPS inhibited ESCs migration from 12 to 36 h (*p* < 0.05) (Figure 6B,C). After ESCs were incubated with LPS for 24 and 36 h, AR treatment reversed the inhibitory effect of LPS on cell migration (*p* < 0.05) (Figure 6B,C).

Impairment of wound healing is a hallmark of age‐induced dysfunction of skin.^22^ Compared with 2M mice, 24M mice showed slower wounds healing from d3 to d9 with statistically difference (*p* < 0.01). The decrease in wound closure rate in 24M mice skin was improved by AR (40 mg/kg) treatment from d3 to d7 (*p* < 0.05), indicating the protective effect of AR on the wound healing function of the skin (Figure 6D,E).

## 4 DISCUSSION

It is worth noting that an increased level of inflammation response has been widely reported during human ageing, which can induce damage to cell function and longevity.^29^, ^30^ SASP and its mediated inflamm‐ageing are important mechanisms in ageing.^31^ As the main source of oxidative stress in cells, mitochondria are closely related to inflammation and apoptosis.^32^, ^33^ Accumulating evidence suggests a strong link between mitochondrial dysfunction and ageing‐associated diseases.^34^ A large amount of evidence suggests that AR can play a role in a variety of diseases by regulating the function of mitochondria.^35^, ^36^ However, its influence on mitochondria in the process of skin ageing and its effect on wound healing in aged skin remains to be further studied.

We found that the inflammatory factors which elevated with age were mainly distributed in the basal layer of the epidermis, probably owing to the epidermis being the outermost layer of the body, which is affected not only by endogenous ageing but also photoageing. Located in the basal layer of the epidermis, ESCs are the key in maintaining epidermal homeostasis and the re‐epithelialization of wounds, hence the key to the treatment of inflamm‐ageing. It is reported that adiponectin can restore the defective hematopoietic cell proliferation of obese and adiponectin‐deficient mice by suppressing bone marrow inflammation.^37^ Adiponectin can also act on the blood vessels to inhibit macrophage inflammation.^38^ Here, we found that AR showed a capacity of eliminating the elevated secretion of inflammatory factors, thereby partially delaying inflamm‐ageing in the skin of aged mice in vivo. Also, AR showed a protective effect against inflammation factors on LPS‐treated ESCs in vitro. Undoubtedly, the increased apoptosis in the basal layer of aged skin or in ESCs incubated with LPS was also inhibited by AR, manifested as lower expression of cleaved caspase‐9, cleaved caspase‐3 and Bax.

Among the most classic pathways to induce apoptosis in cells, the Caspase family plays an important role. The cascade activation of caspase can be induced with the structural abnormalities and dysfunction of mitochondria. To probe the underlying mechanism, we focused on the effects of AR on the mitochondria of aged skin and ESCs. Mitochondria of basal layer cells in aged skin were deteriorating, manifested by swelling and rupture in morphology and reduced ATP synthesis in function, which were partially restored by AR treatment in vivo. Consistent with these results, AR treatment also rescued the shape, basal OCR and ATP production of mitochondria in LPS‐treated ESCs. Here, we demonstrated the protective effect of AR on the mitochondrial morphology and function of ESCs and aged skin. This may account for the reduced level of inflammatory factors and apoptosis after AR treatment.

Therefore, we tried to find the key regulator in AR reversing the damage of mitochondrial morphology and function against the inflamm‐ageing of skin. Drp1 is a cytoplasmic protein, which acts as a key regulator in regulating mitochondrial division. Under stress, Drp1 can be recruited to the outer mitochondrial membrane and thus trigger the division of mitochondria.^39^ Studies have shown that excessive mitochondrial translocation of Drp1 not only triggers increased mitochondrial fragmentation, but also leads to increased mitochondrial ROS generation. Meanwhile, Cytc was released to the cytoplasm, therefore triggering inflammatory response and apoptosis.^40^, ^41^, ^42^ Inhibiting the Drp1 can ameliorate ischaemic renal injury by blocking mitochondrial fission.^43^ However, the role of Drp1 in the inflamm‐ageing of skin is still unclear. The role of AR in regulating Drp1‐mediated mitochondrial division in aged skin has not been reported yet. In this study, we proved that AR could inhibit the mitochondrial translocation of Drp1 under inflamm‐ageing, thereby rescuing the mitochondrial membrane potential and permeability, with morphology and function restored both in vivo and in vitro experiments.

Then, we aimed to elucidate the specific mechanism by which AR regulated Drp1‐mediated mitochondrial division. The function of Drp1 is regulated by phosphorylation, acetylation, glycosylation, etc.^44^, ^45^ Studies have shown that phosphorylation of Drp1 at S616 accelerates its recruitment to the mitochondrial membrane, while phosphorylation at S637 hinders this process.^46^ We found that the phosphorylation of Drp1 at S616 increased while the phosphorylation at S637 decreased with the incubation of LPS in ESCs in vitro. AR treatment could enhance the phosphorylation of Drp1 at S637 in ESCs with little effect on S616. This finding inspired us to probe further. According to reports, AMPK, PKA/GSKIP/GSK3β, STAT2, etc., can regulate the phosphorylation of Drp1 at S637. The AMPK pathway has also been proven to be the classic downstream of AR/AdipoR.^47^ Therefore, we used Dorsomorphin, a selective inhibitor of AMPK for further verification. The protective effect of AR was abolished by Dor on mitochondrial membrane permeability, inflammatory factors and apoptosis. These studies indicated that regulating the phosphorylation of Drp1 at S637 was a potential therapeutic target for protecting the mitochondria under inflamm‐ageing, and AR depended on AMPK to suppress Drp1‐mediated mitochondrial damage.

In previous studies, adiponectin was identified as a regulator of murine cutaneous wound healing by promoting keratinocyte proliferation and migration via the ERK signalling pathway.^18^, ^48^ Also, adiponectin can help maintain skin barrier homeostasis by enhancing lipid synthesis and differentiation of human keratinocytes through SIRT1 and nuclear hormone receptor signalling pathway.^49^ Here, we found that in addition to protecting the morphology and function of mitochondria, AR improved the proliferation and migration of ESCs with the presence of LPS in vitro. Wound healing in aged skin was also accelerated by AR, probably owing to the reduced inflammatory response and improved proliferation and migration of ESCs. This evidence indicated that AR may be used as a potential drug to accelerate wound healing in aged skin.

This research has the following limitations. First, we only studied the protective effect of AR against the inflamm‐ageing of the skin by regulating the division of mitochondria, but it is not clear how it affects the mitochondria in young skin. Second, this research mainly focuses on the effect of AR on ESCs, but the effect of AR on other cells in the skin such as fibroblasts, vascular endothelial cells, Langerhans cells and dendritic T cells deserves further study. Third, AMPK can regulate the mitochondria in many ways, such as regulating mitochondrial metabolic activity through PGC‐1α and mitochondrial autophagy. However, we only explored the impact of AMPK on Drp1. Meanwhile, besides AMPK, AR may also function in other signalling pathways. Further research is urgently needed to detect more details. At the same time, more research is needed to evaluate the pharmacokinetics and long‐term effects of AR in vivo. Due to the important role of AR in metabolic regulation, it is meaningful to explore the effect of AR on wound healing in diabetic or obese patients. Finally, we have a problem that cannot be ignored: using LPS to simulate ESCs under the state of inflamm‐ageing is a crude in vitro experimental model for the ageing skin, which could not reflect the actual situation of skin ageing well, and this urgently needs to be further improved.

In addition, we should also note that this new treatment method will face many challenges before starting a clinical use. (1) Besides age, the inflamm‐ageing of skin is also affected by individual factors such as gender, nutritional status and type of work. The animal model used in this experiment cannot fully simulate the degeneration of human skin during the ageing process. This requires us to further verify the effectiveness of AR with animals and cells models that are much closer to the actual skin changes during human ageing. (2) The mice used in this experiment are healthy mice of different ages. Yet, elderly patients in clinical practice often have comorbidities such as diabetes, cardiovascular diseases, or tumours. Besides, many chronic and incurable wounds in elderly patients are secondary to tumour radiotherapy, chemotherapy, surgery. Before receiving wound‐associated treatment, many other drugs have been used. Therefore, we must consider whether the use of AR will affect the treatment of other comorbidities in elderly patients.

In summary, aged skin shows excessive secretion of TNF‐α, IL‐6 and IL‐1β. The mitochondrial translocation of Drp1 caused excessive mitochondrial division, accompanied by the ensuing increased production of mitochondrial ROS and inflammatory factors, decreased mitochondrial membrane potential and increased mitochondrial membrane permeability. Cytc is released into the cytoplasm and may play partially in inducing caspase‐mediated apoptosis. AR can depend on AMPK to regulate the phosphorylation of Drp1, thereby inhibiting the excessive division of the mitochondria, thus exerting a protective effect on the skin and ESCs. Our research provides an opportunity to develop preventive and therapeutic drugs to treat SASP and inflamm‐ageing‐associated skin diseases in which mitochondrial dysfunction plays an important role.

## 5 ETHICS APPROVAL AND CONSENT TO PARTICIPATE

This study was performed in compliance with the principles of the Helsinki Declaration and Guidelines for the Care and Use of Laboratory Animals of the Chinese Institute of Health. All procedures using animals were approved by the Animal Research Committee and Ethics Committee of General Hospital of PLA. The use of human foreskin was approved by the Ethics Committee of General Hospital of PLA. We obtained the written informed consent from all the patients participated in this study.

## COMPETING INTERESTS

The authors declare that they have no competing interests.

## CONFLICT OF INTEREST

The authors declare that the research was conducted in the absence of any commercial or financial relationships that could be construed as a potential conflict of interest.

## AUTHOR CONTRIBUTIONS

C.S. conceptualized the study and designed experiments. J.S. and Y.N. wrote the manuscript. C.S. critically reviewed the manuscript. J.S., X.L. and W.Z. performed experiments, collected and analysed the data.

## Data Availability

Further information and requests for reagents may be directed to and will be fulfilled by the Lead Contact: Chuan'an Shen (shenchuanan@126.com).
